# An empirical study of continuous use behavior in virtual learning community

**DOI:** 10.1371/journal.pone.0235814

**Published:** 2020-07-17

**Authors:** Wei Liu, Yue Wang, Zhixin Wang

**Affiliations:** Business School, University of Jinan, Jinan, China; West Pomeranian University of Technology, POLAND

## Abstract

A virtual learning community (VLC) is a learning-based integrated platform based on a network sharing mechanism. In a VLC, users are not only the extractors of knowledge results but also the source of the platform knowledge base. The continuous knowledge sharing and communication of VLC users promote the sustainable development of the community. Studying the continuous use behavior of users in the VLC for the sustainable development of the community is of great importance. This study introduces the perceptual normativeness, perceptual enjoyment, perceptual interactivity, and perceptual value variables on the basis of the extended expectation confirmation model of the information system continuance model. After checking and correcting the prediction questionnaire twice, we adopted the "Questionnaire Star" network system to distribute the questionnaire and conducted offline questionnaire surveys of borrowers of Shandong University Library, University of Jinan Library, and Shandong Library. A theoretical model of the continuous use behavior of users in the VLC is constructed using the structural equation model. The survey data were empirically analyzed to explore the influencing factors and mechanism of the continuous use behavior of users in the VLC. Interaction perception, value perception, and perceptual normativeness exerted a significant positive impact on user-perceived usefulness and expectation confirmation, whereas perceptual enjoyment had little effect on perceived usefulness. This study also summarized the relationship between users’ continuous use behavior, continuous willingness and perceived usefulness, expectation confirmation, satisfaction, self-efficacy, and contributing factors in the VLC on the basis of the data results. Suggestions for solving the identified problems are put forward.

## Introduction

The Virtual Learning Community (VLC) is an online community based on interest and knowledge sharing [1–2]. Therefore, user-generated content is an important factor in the survival and development of virtual communities. Meanwhile, the mainstream opinions in a community and the core members of such community exert a major impact on the learning behavior of other users [3]. It has resulted in continuous user behavior in VLC, which refers to the communities’ capability of constantly attracting and protecting users [4]. It promotes the development and promotion of VLC. Empirical research shows that VLCs not only present small-world network features whose core is marginalization [3, 5, 6], but also have the strong and weak relationships between subjects’ social networks [7–8]. These all lead to knowledge flow in VLC. Knowledge sharing, trust, and flow are the key factors for users to continue using VLC. At present, VLCs have become a hot issue in distance education research. These communities provide an opportunity for the study of distance education and online learning. In addition, VLCs provide a good platform for the effective training of students’ high-level abilities, the improvement of their collaboration and communication skills, and the optimization of knowledge building dimensions [9]. According to relevant literature analysis, many scholars are currently engaged in extensive research on VLC in the fields of education, psychology, sociology, computer science, behavior science, etc., and provide a lot of theoretical research on VLC, such as network construction and collaborative development, academic exchange Factors and learner characteristics [10–12]. However, the research on the continuous use behavior of users in VLCs and the sustainable development of these communities is not deep enough. The short-term popularity of learners, the continuous loss of community resources, and the low degree of dependence of learners on communities will affect the sustainable development of VLCs [13].

At present, the society is rapidly advancing into a new era of information. The awareness of lifelong learning has been deeply rooted in the hearts of the people. The effective flow of social resources has increased people’s desire for new knowledge as China’s openness continues to increase [14]. As a kind of communication learning platform, VLCs have become an important tool for knowledge construction and learning of the entire nation because they are not restricted by geographical conditions and they comprise condensed collective wisdom. At present, China’s education informatization reform has already reached a certain scale, education methods have been continuously innovated, and the demand for quality educational resources has gradually increased [15]. The continuous advancement of the “One Belt, One Road” national strategy, which is the abbreviation of "the Silk Road Economic Belt" and "21st-Century Maritime Silk Road" and was a cooperation initiative to achieve economic cooperation, political mutual trust, and cultural tolerance, has brought unprecedented opportunities for the development of VLCs based on online media. The inclusiveness, openness, quality sharing, and mutual experience of education have become major driving forces for the common development of VLCs in various countries under the “One Belt, One Road” project [16]. The establishment of educational communities have also become a common vision for countries and regions involved in the project. Countries have jointly established a community of cultural inclusion interests centered on the “Belt and Road.” The objective is to create a group of education-centered academic exchange platforms that bring together science and technology and economies in various countries and regions, geography, culture, and other areas of knowledge. Another objective is to attract high-end scholars and experts from various countries and regions to carry out academic exchanges and knowledge collisions at different levels. In this environment, VLCs need to not only meet the growing demand for citizen network learning in China but also ensure the security, reliability, and sustainability of each learning community. VLCs should also complete the interconnection between countries and international docking and perform their expected functions in national online education and international education cooperation.

VLCs feature user autonomy, through which users can choose the knowledge they need. This kind of autonomous and selective learning communication refers only to users’ initial decision-making choices, and whether a learner can complete the whole process remains to be verified. The outstanding problems in the actual development of VLCs include high user churn rate, low user dependence, low user stickiness, and withdrawal of community members [17]. This phenomenon is unfavorable for the knowledge sharing and learning exchange of community members and leads to economic and time losses for the previous investment. Most of the current research on user churn rate is focused on traditional distance education, which is different from the continuous use behavior of users in VLCs. Traditional distance education learning is generally an organized collective learning process [18] and is not a completely active choice learning process. The behavior of users in a community is completely autonomous. The ultimate goal of the construction and development of VLCs is the continuous expansion and use of quality education and learning resources by users in the communities. Other goals include the popularization of quality education resources and the promotion of online education [19]. Therefore, the present study intends to build a research model of the continuous use behavior of users in VLCs on the basis of relevant learning behavior theory and to explore the mechanism of users’ continuous use behavior and its influencing factors [20]. The purpose is to provide theoretical guidance and practical advice for the sustainable development and construction of VLCs and to promote online learning and international education cooperation in the world.

## Theoretical basis of research

The research on continuous use behavior mainly uses the technology acceptance model (TAM), the theory of planned behavior (TPB), and the expectation confirmation model of information system continuance (ECM-ISC) as the theoretical framework.

The TAM was proposed by Davis to study users’ theory of the adoption and acceptance of information systems (ISs) [21]. Bhattacherjee believed that the success of IS depends not only on the initial use of users but also on their continued use [22]. The main theoretical framework of the TAM is based on three variables of rational behavior theory, namely, behavioral attitudes, behavioral intentions, and actual behaviors; and sets the external variables of perceived usefulness and perceived ease of use [21]. The TAM has good interpretation and prediction ability for user adoption and application behavior in ISs, but it also has certain limitations. For example, the social impact process is not considered in the model. In order to improve the ability of TAM to predict and explain the behavior of users of information systems, scholars such as Park (2014) [23] and Tarhini et al (2014) [24]. Have extended and improved the model. The expanded and modified TAM model is comprehensive and has gradually become a typical theoretical basis and support for users of ISs. This model is widely used in related fields of user behavior research in ISs.

The TPB was first proposed by Ajzen as an extension of rational behavior theory to explain behaviors that are not completely controlled by individual will. Meanwhlie, TPB believes that human behavior is the result of thoughtful planning. Ajzen’s publication of the theory of “Planned Behavior” in 1991 is a sign of the maturity of the theory. The theory of planned behavior includes five elements: Attitude, Subjective Norm, Perceived Behavioral Control, Behavior Intention, and Behavior. The application of TPB can be divided into three categories: predicting individual behavior, expanding into the field of new media, and cross-cultural application of TPB. Of course, TPB also has certain limitations, such as the object of TPB must be individual rational behavior; the elements of the TPB model must be aimed at the same object and belong to the same level.

**Fig 1:**
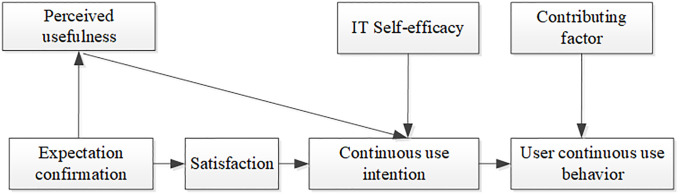
Extended information system continuous use model.

The ECM-ISC was proposed by Bhattacherjee (2001) on the basis of the expectation confirmation theory (ECT) [25] to explain users’ continuous use behavior and abandon the use behavior model. The research on the ECM-ISC focuses on the continued willingness and behavior of users who have not discontinued use after the initial adoption of ISs; this model is widely applicable to various IS environments [26]. The domestic application of the ECM-ISC began in 2006. The model was subsequently applied to various fields. Limayem (2007) improved the model based on the ECM-ISC by extending the dependent variable from original willingness for continuous use to continuous use behavior [27]. The ECM-ISC introduces the perceived usefulness in the TAM model. The influence of perceived ease of use on continuous use behavior is gradually weakened in the later stage when the IS is continuously used. Hence, the perceived ease of use is not introduced. Bhattacherjee believed that the core of judging the success of the implementation of any IS is whether the user has a continuous use intention and whether it depends on the IS; such assessment is not limited to the subjective selective behavior of the initial user. The ECM-ISC focuses on the failure to generate high user behavior when the user selects a particular IS. In the use of the IS, the three elements that influence continuous use are “user satisfaction,” “perceived usefulness,” and “expectation confirmation.” Satisfaction has a positive impact on users’ willingness to continue to use. The expectation of recognition affects users’ willingness to use by affecting users’ perceived usefulness and satisfaction. Since its introduction, the ECM-ISC has been widely used by many researchers [28]. The model has been proved to have certain interpretation and prediction capabilities. However, Bhattacherjee explained that stopping the research on users’ willingness to use is not enough because users’ willingness to use will eventually be transformed into user stickiness; hence, the ECM-ISC has been revised and expanded [29] (Fig 1).

The expanded ECM-ISC introduces two variables, namely, computer self-efficacy and contributing factors in perceptual behavior control theory. The perceived usefulness in the original model significantly affects users’ satisfaction. Perceived usefulness is a correct reflection of users’ cognition. It also exerts a certain impact on users’ initial choice and continuous use. Users’ willingness to continue to use has a positive impact. The extended model can provide a more detailed explanation and prediction of the continuous willingness and behavior of IS or IT users compared with the original ECM-ISC.

## Research model and research hypothesis

**Fig 2:**
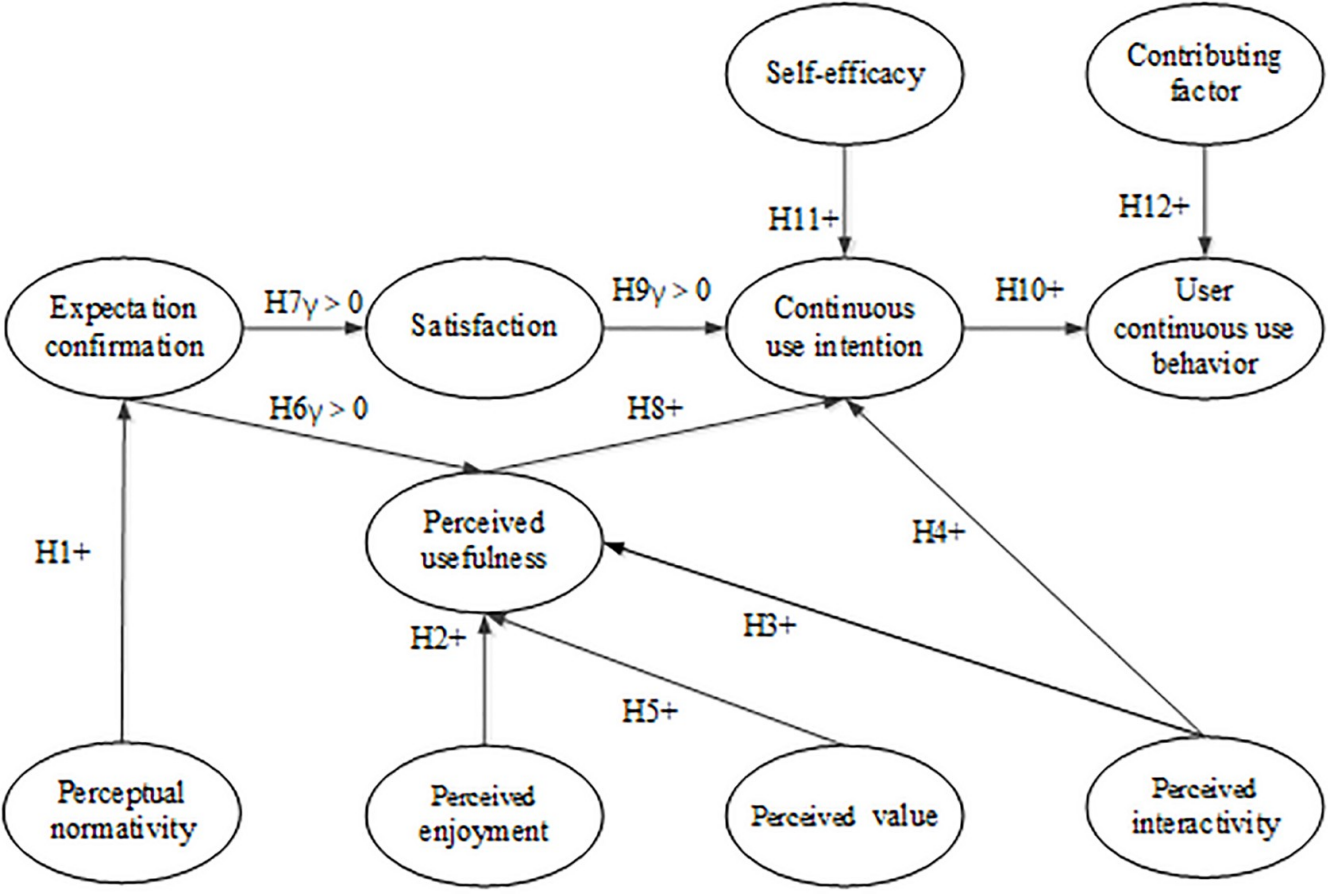
VLC users’ continuous use behavior research model. “+” Positive influence; “γ” Correlation coefficient.

VLCs are inevitable outcomes of the development of educational technology to a certain extent. VLCs are used to solve the common needs of learners from different fields and regions, and share network space through certain technical means and network standards. Many scholars have conducted relevant research on this. However, they are now more focused on the development and construction resource creation, learning evaluation, and the impact factors of user composition [30]. The research on users’ continuous use behavior from users’ points of view is limited. In particular, the research on the important influencing factors and impact mechanisms of initial acceptance and continuous use of VLC users has not received sufficient attention from domestic research. Perceptual normativeness, perceptual enjoyment, perceptual interactivity, and perceptual valuehers. To this end, the present study uses the extended ECM-ISC as the theoretical framework, along with VLCs and their usage characteristics. Moreover, the present study introduces the variables of perceptual normativeness, perceptual enjoyment, perceptual interactivity, and perceptual value. A research model of the continuous use behavior of VLC users is subsequently constructed, As shown in Fig 2.

The research on user behavior toward ISs in the current academic field is sorted from the perspective of development, and the research on users’ continuous use behavior is mostly based on the basic theoretical model. The research variables in the original model are refined and modified according to the different research objects so that the constructed model can conform to specific situations. The research field has no established evaluation index system for VLCs. Hence, this work combines the domestic and foreign literature on evaluation index systems for VLCs [31–33]. Referring to existing models and theories, this study proposes to discover VLCs by adding four dimensions based on the extended ECM-ISC, namely, perceptual normativity, perceptual enjoyment, perceptual interactivity, and perceptual value.

Perceptual normativeness is mainly used to test the operability, stability, security, human–computer interaction, and interface friendliness of a VLC support system or platform. Perceived value includes the perception of users’ self-contribution and the value of the community’s knowledge. The purpose of users in participating in VLCs is to acquire knowledge to enhance themselves. The contribution of users to community knowledge is the basis of community development. Perceived enjoyment is the degree of enjoyment of a user during use and is not related to the results of the use. Perceptual interactivity is mainly used to test interactions in a VLC. In communication interaction, users in a VLC freely exchange views and opinions. Moreover, users acquire the required knowledge through effective communication and collaborative learning, and they integrate such knowledge into their own knowledge structure. In addition, the interactivity in a VLC includes the interaction between users and the platform. This kind of interaction refers to whether the user can get timely feedback from the platform when the user faces problems in the process of using it. Perceptual normativeness, perceptual enjoyment, perceptual interactivity, and perceptual value exert an important influence on users’ expectation degree of recognition, perceived usefulness, and continuous use intention in the VLC. Therefore, the following hypotheses are made:

**Hypothesis 1 (H1)**. The perceptual normativeness of the VLC positively affects users’ expectations.
**Hypothesis 2 (H2)**. The perceived enjoyment of the VLC positively affects the perceived usefulness of users after use.
**Hypothesis 3 (H3)**. The perceived interactivity of the VLC positively affects the perceived usefulness of users after use.
**Hypothesis 4 (H4)**. The perceived interactivity of the VLC positively affects users’ continued use intention.
**Hypothesis 5 (H5)**. The perceived value of the VLC positively affects the perceived usefulness of users after use.

Expectation confirmation theory reflects users’ cognitive level to a certain extent. Users will have certain expectations for a VLC before using it. After using the VLC, the users will have certain knowledge of the VLC. Users will compare their expectation with their knowledge of the VLC after use to form a confirmed behavior. The degree of expectation and confirmation can determine the degree of user satisfaction. If the expectation of community users is high, then the satisfaction is high, and the willingness to use is strong. For the VLC, perceived usefulness is the prediction of the degree to which a user’s learning objectives are completed in the VLC. The *perceived usefulness* in the extended ECM-ISC is a reflection of users’ cognitive beliefs and is thus different from the perceived usefulness of a single initial contact IS or IT in the TAM. User satisfaction is the degree to which users are satisfied. Users’ satisfaction with a VLC mainly comes from the comparison between their pre-use expectations and their post-use cognition. Moreover, users’ satisfaction significantly affects their willingness to use and their continuous use behavior. Therefore, the following hypotheses are made:

**Hypothesis 6 (H6)**. The degree of Expectation confirmation in the VLC is positively correlated with perceived usefulness.
**Hypothesis 7 (H7)**. The degree of Expectation confirmation in the VLC is positively correlated with user satisfaction.
**Hypothesis 8 (H8)**. The perceived usefulness of users in the VLC positively affects their continued use intention.
**Hypothesis 9 (H9)**. Users’ satisfaction in the VLC is positively correlated with their continued use intention.

Rational behavior theory holds that users’ subjective behavior intention in the IS has a direct impact on their actual behavior. Behavioral intention is a user’s willingness to perform certain behaviors and is a measure of behavioral tendencies. The continuous use behavior of users in a VLC refers to their initial acceptance and adoption of the VLC for a period of time to use it for their own learning or teaching activities. In many cases, users’ behavioral intentions and behaviors lack a one-to-one correspondence. However, multiple behavioral intents can lead to certain behaviors. To effectively explain the individual differences of system users and the influence of external factors on learners’ behavioral intentions and individual behaviors, Bhattacherjee incorporated the variables of self-efficacy and contributing factors in the theory of perceived behavioral control under social psychology [34]. Self-efficacy is a subjective evaluation of users’ independent behavior, and it mainly reflects the intrinsic ability of an individual. Contributing factors refer to learners’ perception of controllable external resources and reflect the availability of external resources [35]. After the initial use of the IS, users choose whether or not to continue using it according to their self-efficacy [36]. After using the VLC, users may intend to give up its use directly because of their knowledge and learning ability. Facilitating factors can directly determine the continuous use behavior of users in the community. If users want to continue learning in the community but lack internet tools or networks, then they give up the behavior of continuing to learn. Therefore, the following assumptions are made:

**Hypothesis 10 (H10)**. The continued use intention of users in the VLC positively affects their continuous use behavior.
**Hypothesis 11(H11)**. The self-efficacy of users in the VLC positively affects their continued use intention.
**Hypothesis 12 (H12)**. The enabling factors of users in the VLC positively affect their continuous use behavior.

## Research design and data analysis

### Questionnaire design and data collection

We used a questionnaire to collect the data needed for this study. First, the related variable scales from the domestic and foreign literature were reviewed on the basis of the research content of this study. The scale is shown in Table 1.

**Table 1:** Questionnaire measurement indicators and sources.

Latent construct	Item	Measurement standard	Sources
Perceptual Normativeness (PRN)	PRN1	The operating system of the VLC platform has good performance and stable operation.	Delone&Mclea (1992) [37]; Zeithaml&Malhotra (2005) [38].
	PRN2	The interface layout of the VLC platform is simple and reasonable, easy to operate, and the interface design is beautiful and generous.	
	PRN3	The VLC platform is fully functional and easy to use.	
	PRN4	The VLC system is safe and reliable, and pays attention to the privacy protection of users.	
Perceived Enjoyment (PRE)	PRE1	Using the VLC has brought me a lot of joy.	Chun-Hua,Hsiao (2016) [39]; Julian K. Ayeh (2013) [40].
	PRE2	The content on the VLC makes me very interesting.	
	PRE3	My interaction with other users makes me feel happy and content.	
Perceived Interactivity (PRI)	PRI1	I can take the initiative to share new knowledge with my friends in the VLC.	Iivari (2014) [41]; Parasuraman (2005) [42].
	PRI2	I would like to keep in touch with friends in the VLC.	
	PRI3	I ask questions to get responses from others in the VLC.	
	PRI4	I have easy access to learning resources in the VLC.	
	PRI5	I can easily post ideas in the VLC.	
	PRI6	I can evaluate the knowledge and information provided in the VLC, and share the learning information can be rewarded.	
Perceived Value (PRV)	PRV1	I use a VLC for learning, which strengthens my understanding of relevant knowledge.	Hofbrook (1999) [43]; Sweeney & Soutar (2001) [44].
	PRV2	I use this VLC for learning and I have acquired many skills.	
	PRV3	I use this VLC for learning and improve my learning efficiency.	
	PRV4	I think this VLC provides a direction for continued research.	
	PRV5	I think the knowledge provided by this VLC is instructive in future practice.	
Expectation Confirmation (DSC)	DSC1	After using this VLC, I feel it is better than I expected.	Samar Mouakket (2015) [45]; Thong. (2006) [46].
	DSC2	The service provided by this VLC is better than I expected.	
	DSC3	Most of my expectations for using this VLC have been confirmed.	
Perceived Usefulness (PSU)	PSU1	The learning information on the VLC was helpful to me.	Julian K. Ayeh (2013) [40]; Kim&Chan (2008) [47]; Bhattacherjee (2008) [48].
	PSU2	In the VLC, I easily find the information I need.	
	PSU3	The products and services provided by the VLC have been useful to me.	
	PSU4	The VLC is used to improve my learning efficiency	
Customer Satisfaction (STN)	STN1	All my experiences about using this VLC have made me very satisfied.	Samar Mouakket (2015) [45]; Thong (2006) [46]. Bhattacherjee (2001) [49].
	STN2	I think my decision to use this VLC was correct.	
	STN3	All my experiences about using this VLC have made me very happy.	
	STN4	I am satisfied with the learning results after using the VLC.	
Continuous Use Intention (CNI)	CNI1	I would like to continue using this VLC.	Chun-Hua Hsiao (2015) [39]; Julian K. Ayeh (2015) [50]; Bhattacher Jee (2002) [51].
	CNI2	I plan to continue using this VLC.	
	CNI3	I will often use this VLC in the future.	
	CNI4	I have positive comments about this VLC.	
Continuous Use Behavior (CNB)	CNB1	In the future, I will also use the VLC for learning.	Davis (1989) [52]; Venkatesh (2003) [53]; Limayem (2007) [54].
	CNB2	I will often use VLC in the future.	
	CNB3	I would like to recommend a VLC to friends and relatives.	
	CNB4	I have encountered related problems and I will try to solve them with a VLC.	
Self-Efficacy (SLE)	SLE1	I can use the VLC independently.	Bhattacherjee, Perols (2008) [37].
	SLE2	I use a VLC for learning.	
	SLE3	I am confident in my ability to learn using a VLC.	
Contributing Factor (FCC)	FCC1	I use more energy and time in the VLC.	Liu Luchuan et al (2011) [55]; Zhao Yang et al (2015) [56].
	FCC2	I think the VLC is a bit slow to open.	
	FCC3	People around me treat the VLC positively.	

Moreover, the structure, problems, and options of the questionnaire were determined. The questionnaire was divided into three parts: the explanation of the questionnaire, the test questions and options, and the main information about the respondents. The questionnaire description included the explanations of the questions and related concepts. The test questions were aimed at identifying the scale required by the research from the relevant variable scales collected in the review process. The contents and items of this initial scale were then modified and adjusted on the basis of discussions with tutors and classmates to form a predictive questionnaire. A five-level Likert scale was adopted to design the topic options for the questionnaire. The respondents rated their knowledge and usage of the VLC as follows: strongly agree = 5 points, consent = 4 points, generally agree = 3 points, disagree = 2 points, strongly disagree = 1 point. The last part of the questionnaire covered the personal information of the respondents, including their gender, age, education, and specific occupation.

We carried out a small-scale test of the prediction questionnaire prior to the formal survey to test whether the questions and options in the questionnaire suffer from ambiguity or semantic problems, as well as to ensure the rationality and comprehensiveness of the questionnaire. First, we invited 10 experts in pedagogy research to explain the meanings of the questions in the questionnaires to the pretesters. We corrected the questionnaire on the basis of their questions and potential problems. Second, we randomly selected 35 college students to answer our questionnaire to ensure the reliability and validity of the test scale. We formed our final questionnaire on the basis of the improvements identified.

After the completion of the questionnaire, we conducted the survey using two major methods. The first method was to distribute the questionnaire through the “Questionnaire Star” network system and then promote it using network communication tools such as WeChat and QQ. The second method was through offline visits. The survey subjects were mainly divided into three main categories, namely, the Shandong University Library, the University of Jinan Library, and the Shandong Provincial Library. Highly educated college students are the main users of VLCs. Most of them come from different regions. Their majors are extensive, and they possess a certain understanding of VLCs. In this study, most of the respondents were actual users of VLCs and thus have extensive experience in using these virtual communities. The survey included a wide range of people, with a relatively dispersed age group and certain representativeness. Most of the respondents were public officials working in relevant units, and some were students preparing for career editing, civil service, postgraduate examinations, etc. All of them have the motivation to actively learn. Most of the respondents had experienced online learning and were not new to VLCs. Some of them were even long-term users of VLCs whose needs could be met by these VLCs. The sample selection was critical. Considering the experience of users in using VLCs in the electronic reading rooms of university libraries is important in studying the influencing factors and mechanisms of community user behavior. A total of 380 questionnaires were distributed, and 341 questionnaires were returned; among those returned, 296 questionnaires were deemed valid. The effective recovery rate was 86.8%. Table 2 shows the specific information of the study sample.

**Table 2:** Descriptive statistics of survey sample.

Variable	Project	Quantity	Percent
Gender	Male	134	43%
	Female	162	57%
Age	≤20	18	6%
	20–30	275	93%
	30–40	3	1%
	≥40	0	0
Education background	Specialist	2	0.7%
	Bachelor	167	56.4%
	Master	98	33.1%
	PhD	29	9.8%

### Reliability and validity tests

The reliability test is a test of the credibility of survey data, and it mainly reflects the continuity and stability of survey results. The validity test relates to the level of a given problem measured by a scale. If validity is high, then the measurement scale meets the measurement requirements. In this work, the collected data were sorted and analyzed, and the reliability and validity of the test scale were measured by SPSS 25.0 and Amos 20.0 data statistics software. The credibility of the survey data was tested using the level of Cronbach’s coefficient according to the principle of statistical correlation. Composite reliability (CR) can effectively evaluate the degree of correlation of measured variables and is a commonly used evaluation variable that can reflect the degree of internal consistency of latent construct. Validity tests include test validity and discriminant validity. The average extracted variance (AVE) value reflects the proportion of measurement error in the variance explained by the latent construct. AVE can effectively check the discriminant validity of the scale, and the aggregate validity can be measured by the standard load. Table 3 shows the measurement results.

**Table 3:** Reliability and validity test results.

Latent construct	Item	Standardized Factor Loading	AVE	CR	Cronbach’s α
Perceptual Normativeness (PRN)	PRN1	0.710	0.542	0.824	0.744
	PRN2	0.672			
	PRN3	0.831			
	PRN4	0.721			
Perceived Enjoyment (PRE)	PRE1	0.767	0.5302	0.7716	0.743
	PRE2	0.683			
	PRE3	0.732			
Perceived Interactivity (PRI)	PRI1	0.685	0.563	0.884	0.787
	PRI2	0.731			
	PRI3	0.681			
	PRI4	0.762			
	PRI5	0.725			
	PRI6	0.896			
Perceived Value (PRV)	PRV1	0.715	0.613	0.887	0.832
	PRV2	0.882			
	PRV3	0.739			
	PRV4	0.803			
	PRV5	0.765			
Expectation Confirmation (DSC)	DSC1	0.830	0.5312	0.7708	0.730
	DSC2	0.682			
	DSC3	0.663			
Perceived Usefulness (PSU)	PSU1	0.682	0.518	0.810	0.791
	PSU2	0.781			
	PSU3	0.672			
	PSU4	0.739			
Customer Satisfaction (STN)	STN1	0.732	0.515	0.8094	0.883
	STN2	0.692			
	STN3	0.720			
	STN4	0.726			
Continuous Use Intention (CNI)	CNI1	0.820	0.534	0.819	0.796
	CNI2	0.712			
	CNI3	0.613			
	CNI4	0.762			
Continuous Use Behavior CNB	CNB1	0.652	0.523	0.814	0.707
	CNB2	0.876			
	CNB3	0.632			
	CNB4	0.718			
Self-Efficacy (SLE)	SLE1	0.974	0.632	0.8331	0.790
	SLE2	0.736			
	SLE3	0.637			
Contributing Factor (FCC)	FCC1	0.865	0.6412	0.841	0.847
	FCC2	0.664			
	FCC3	0.857			

Reference [24] Standardized Factor Loading > 0.5, CR > 0.6, AVE > 0.5, Cronbach’s α > 0.5.

Table 3 shows that the Cronbach’s alpha value and the CR value of each latent construct are above 0.7 or 0.8, which is ideal and indicative of the scale’s credible. The standard load of each latent construct is above 0.6, and the AVE value is above 0.5. Hence, the observed variables in the measurement scale can effectively evaluate the characteristics of the latent construct, and the scale validity is good. For the accuracy of the test, the data were further subjected to principal component analysis. Prior to the factor analysis, we determined whether the KMO and Bartlett values of the matrix meet the analytical requirements. The results show that KMO 0.914 > 0.9 and that the spherical test value reaches the significance level of P < 0.001, which indicates good suitability for factor analysis. Through the principal component analysis and the maximal rotation of the variance of the factor load matrix, we extracted 11 factors with eigenvalues above 1 and recorded a variance interpretation rate of 74.1%. In the component matrix after rotation, the load value of all measured variables exceeds 0.5, the cross-variable load value is below 0.5, and the factor structure is clear. These outcomes indicate that the scale has good validity and that the questionnaire is reliable. In sum, the internal consistency of the measurement model is good, and the measurement results are reliable.

### Model testing and analysis

In using structural equations to validate models, the appropriate model fit is the premise of use. The degree of fit of the model is the degree of fit between the research model and the actual sample data. If the covariance matrix implied by the research model is close to the covariance matrix of the sample data, then the fit of the model is satisfactory.

In reference to Marsh, we selected the following for testing: chi-square/degree of freedom (CMIN/DF), comparative fit index (CFI), goodness-of-fit index (GFI), incremental fitness index (IFI), normed fit index (NFI), unconventional fitness index (TLI), and root mean square error of approximation (RMSEA). Table 4 shows the results of the confirmatory fitness test.

**Table 4:** Model fitting indicator values and reference values.

Fit Indices	CMIN/DF	CFI	GFI	IFI	NFI	TLI	RMSEA
Test Result	2.716	0.914	0.939	0.925	0.912	0.920	0.015
Guideline	1~3	>0.9	>0.9	>0.9	>0.9	>0.9	<0.08
Conclusion	Qualified	Qualified	Qualified	Qualified	Qualified	Qualified	Qualified

The results of each indicator test of the model meet the requirements of the reference standard. Hence, the covariance matrix of the survey data is consistent with the covariance matrix of the research model, and the fit of the research model is satisfactory. The model assumptions were analyzed using a structural equation model. Therefore, the test results of the hypothesis in this paper can be obtained, as shown in Table 5.

**Table 5:** Hypothesis results for the structural model.

Path	Hypothesis	Standard Path Coefficient.	S.E.	C.R	P	Results
PRN→DSC	H1	0.45	0.087	5.028	**	Supported
PRE→PSU	H2	0.11	0.358	1.982	0.161	Not supported
PRI→PSU	H3	0.36	0.083	4.243	**	Supported
PRI→CNI	H4	0.31	0.065	4.642	**	Supported
PRV→PSU	H5	0.56	0.051	7.458	**	Supported
DSC→PSU	H6	0.29	0.042	6.847	*	Supported
DSC→STN	H7	0.39	0.056	6.931	**	Supported
PSU→CNI	H8	0.51	0.073	6.856	**	Supported
STN→CNI	H9	0.43	0.058	7.279	**	Supported
CNI→CNB	H10	0.33	0.067	4.625	**	Supported
SLE→CNI	H11	0.14	0.360	0.319	0.749	Not supported
FCC→CNB	H12	0.41	0.079	5.045	**	Supported

*p < 0.05;

**p < 0.01.

**Fig 3:**
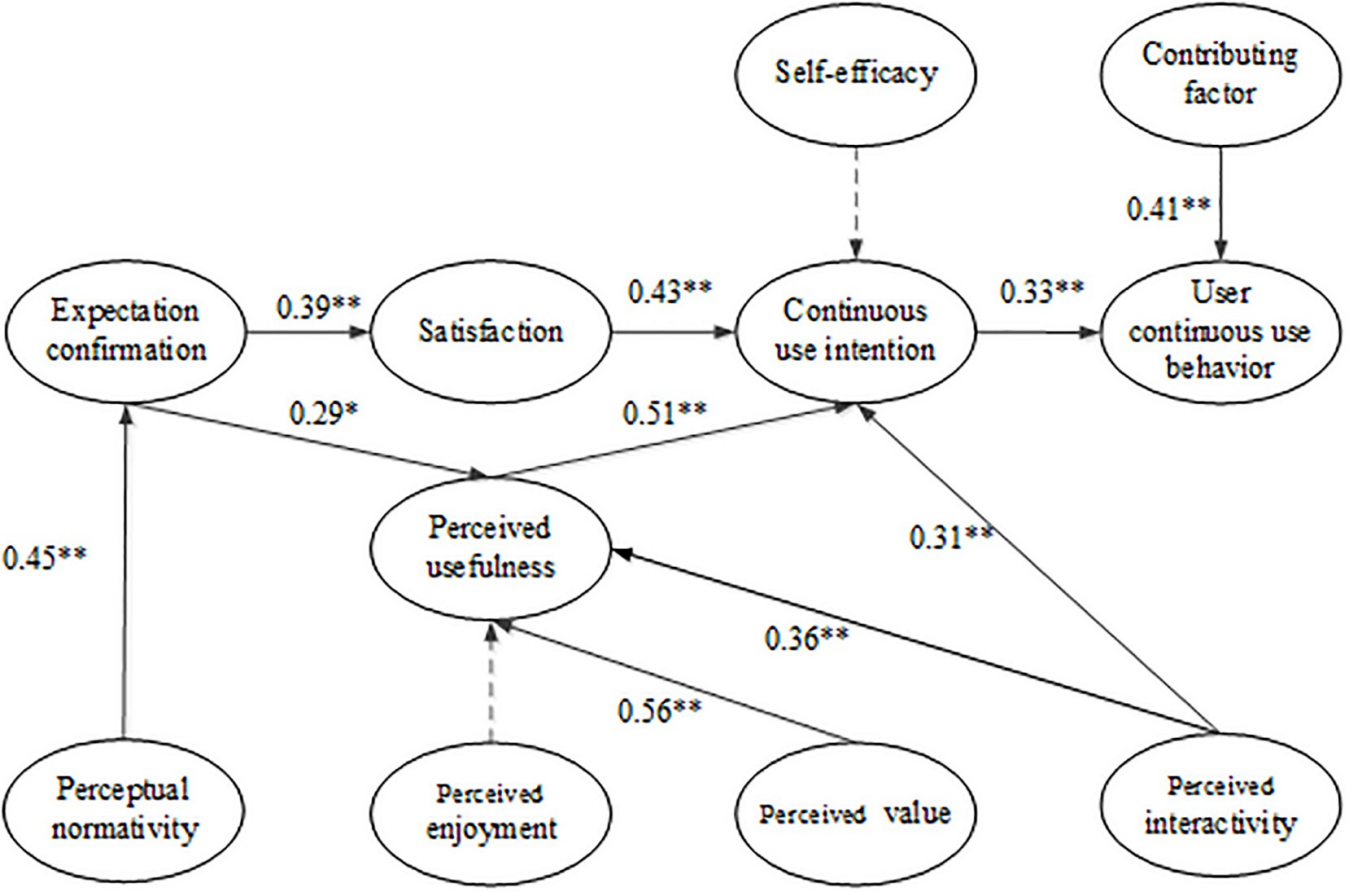
Diagram of structural equation model. *p < 0.05; **p < 0.01; Dotted line indicates non-significance.

In order to see the influence between different variables, we have drawn a research model diagram. Fig 3 shows the test results.

Fig 3 shows that 10 of the 12 hypotheses are verified. Only Hypothesis 2 and Hypothesis 11 fail to pass.

## Discussion

The main relationship between the variables in the structural model path map is discussed as follows.

1. In the VLC, the normativeness of user perception positively affects users’ expectation confirmation, which further affects users’ perceived usefulness; this result is consistent with the interpretation of user acceptance and acceptance behavior in the TAM [57–58]. The first perception of users in utilizing the VLC for the first time comes from the normative nature of the system. Improving the standardization of the system will have a direct impact on users’ confirmation. Among the PRN observation variables, the issue of platform security PRN3 has the most significant impact on the platform (0.87), indicating that the most concerning about the VLC norms is the security of the community. This issue is due to the problem of cybercrime in recent years. Users have a strong sense of prevention. Once the personal information used by VLC users in the registration process is leaked, such information is likely to become the target of criminals. Developers and operators in the VLC should take certain measures to strengthen the protection of the personal information of community users, avoid related losses, and build trust among users. The excellent normative support of the VLC can stimulate users’ potential use awareness and improve users’ expectation of recognition. Therefore, the community should innovate the platform function modules, enrich the learning style, and change thinking approaches to improve the front-end experience of VLC users as a whole.
2. In the VLC, the perceptual value of users positively affects their perceived usefulness while their perceptual enjoyment has no significant effect on perceived usefulness. This finding indicates that some of the students surveyed in this study have rich experience and knowledge in using the VLC and that they will make rational choices according to their own needs. The learning community is different from virtual games, shopping, and other communities. The learning community is a collection of learners who share a common learning language and interest in learning. In such community, their beliefs are strengthened when they face a variety of choices. VLC users are mainly concerned about the exchange and sharing of resources and knowledge in the learning process and the improvement of learning and cognitive ability rather than community entertainment. Therefore, the community should focus on improving the quality of resource services and resources to stimulate users’ continuous use behavior. With regard to the perceptual value of community users, users pay more attention to the value of acquiring knowledge than to the value of self-contribution. In the current information-driven society, interpersonal communication and interactive learning rely heavily on network terminal tools. Such dependence is not conducive to the formation of values and sociality, which is achievable through certain offline activities. Therefore, the VLC needs to organize offline activities on a regular basis to improve the core literacy of users. At the same time, it should strengthen external cooperation, use external forces to achieve overall improvement, encourage users to actively participate in meaningful activities, and cultivate users’ sociality and values. The perceptual value of users is closely related to the degree to which their needs are met. In response to the different characteristics and needs of different users, the community should provide diversified, personalized, and multi-level resource sharing and communication services. New modes of construction and development in the era of knowledge fragmentation are also necessary.
3. The perceptual interactivity of the VLC positively affects users’ perceived usefulness, which in turn influences users’ continuous use behavior. The perceptual interactivity of the VLC positively affects the user's perceptual usefulness and continued use intention. The user's perceptual usefulness also positively affects continuous use intent At the same time, perceptual interactivity indirectly affects users' continuous use intentions through their perceptual usefulness. The interaction between users in the VLC creates a number of connections that are intertwined to form an interactive network. If many interactions exist in the network, users’ perception of the interactions and the connection in the network are strengthened. When this connection is strong enough, the perceptual interactivity affects users’ continuous use behavior and positively influences their intended use. In the research, we found that after a user accepts the VLC, the low-quality interaction for a period of time not only reduces users’ attraction and hinders the development of the community but also causes the interactor to become a potential user of community knowledge to a certain extent. The interaction bias has a direct positive impact on users’ continuous use intention. This finding shows that when the perceptual interactivity reaches a certain standard of user requirements, it can directly affect the intention to use regardless of its quality. This finding also shows that the acceptance of the interaction is relatively high after the user accepts the VLC. In the interaction type, the interaction between users (0.82) is almost as important as the interaction between the user and the system (0.79). The communication and sharing of knowledge in the VLC are based on user interaction. Quality user interaction is the premise of community knowledge transfer. However, providing timely feedback on relevant suggestions and questions raised by users is also important. Timely feedback reflects users’ perceived usefulness and enhances their continuous use intention. Hence, high-quality system software services can enhance user experience. Frequent interactions between learners in the VLC can enhance user loyalty and strengthen mutual trust. When the trust degree of people in the VLC is high, the enthusiasm of users to exchange knowledge in the community will be greatly improved. On the contrary, weak perceptual interactivity will have a negative impact on users’ perceived usefulness. When sharing knowledge, users may worry that their content is negatively affected by irresponsible malicious comments. Such issue is not conducive to VLC resource construction and user perception.
4. In the VLC, users’ expectation degree of recognition has a significant impact on and is positively related to perceived usefulness and satisfaction. This finding is in line with expectation confirmation theory, the interpretation of satisfaction, and perceived usefulness in the ECM-ISC. Moreover, the finding verifies the content of the hypothesis. At the same time, this conclusion shows that in the VLC, if the degree of expectation is high, then the satisfaction and perceived usefulness of users is high. The key to improving user satisfaction and perceived usefulness is to increase user expectations.
5. In the VLC, user satisfaction has a significant impact on continuous use intention, and the effect of self-efficacy on users’ continuous use intention is not significant. This finding shows that users’ focus in their use of the VLC is on whether the community can achieve their intended goals, learning resources, and sharing value. Users’ learning willingness and efficacy, value goals, and mental models will be satisfied and developed and they will be willing to continue their use only after experiencing actual learning through the VLC. Self-efficacy is a subjective judgment. After adopting and accepting the VLC, users may change their intention to continue using the learning objectives, learning effects, learning time, and their own knowledge and skills. The probability of such a change is high due to the subjectivity of self-efficacy, resulting in its insignificant impact on user’s continuous use intention.
6. In the VLC, users’ continuous use behavior is significantly affected by contributing factors and users’ continuous use intention. The contributing factor of the impact on users’ continuous use behavior (0.41) is higher than that of continuous use intention (0.33). However, users 'self-efficacy has a significant positive impact on users' intentions of continuous use, which is limited by their own knowledge and learning ability, and has the intention of giving up continuous use. This result shows that users’ perception of external resources can directly affect their behavioral retention; in subjective knowledge, the latter’s influence exceeds that of the former.

A practical incentive system should be developed to encourage users’ continuous us of the community. The system should pay attention to user feedback, take feasible and timely measures, and offer a free and open platform application environment. The use conditions of the system should be optimized to create a good atmosphere for users.

## Conclusion

Aimed at the continuous use behavior of users in VLCs, this study utilizes the ECM-ISC proposed by Bhattacherjee (2008) as the basic framework structure through the analysis of relevant literature and theories. On the basis of a mature research literature scale, a new IS model is introduced. Variables comprise the model of users’ continuous use behavior in the VLC. They could serve as a reference for the subsequent research on the continuous use behavior of VLC users. In the use of a covariance structure model for empirical testing, the model interpretation rate reached 74.1%, which confirmed the research hypothesis. Although the test model is in good condition, the research results are limited to the scope of the Shandong University Library, the University of Jinan Library, and the Shandong Provincial Library. They also involve a limited sample size. In future research, the capacity and randomness of the sample will be increased to obtain superior results. The follow-up research will examine the changes in the willingness and behavior of users in their later use of the VLC. The mechanism of learning motivation on willingness and behavior is also studied.
